# Supplementary material for: MRE11 and TREX1 control senescence by coordinating replication stress and interferon signaling
Source: Nat Commun. 2024 Jun 26;15:5423. doi: 10.1038/s41467-024-49740-w (PMC11208572; doi:10.1038/s41467-024-49740-w)
Supplement: Supplementary file 1 — Supplementary Information [file 41467_2024_49740_MOESM1_ESM.pdf]

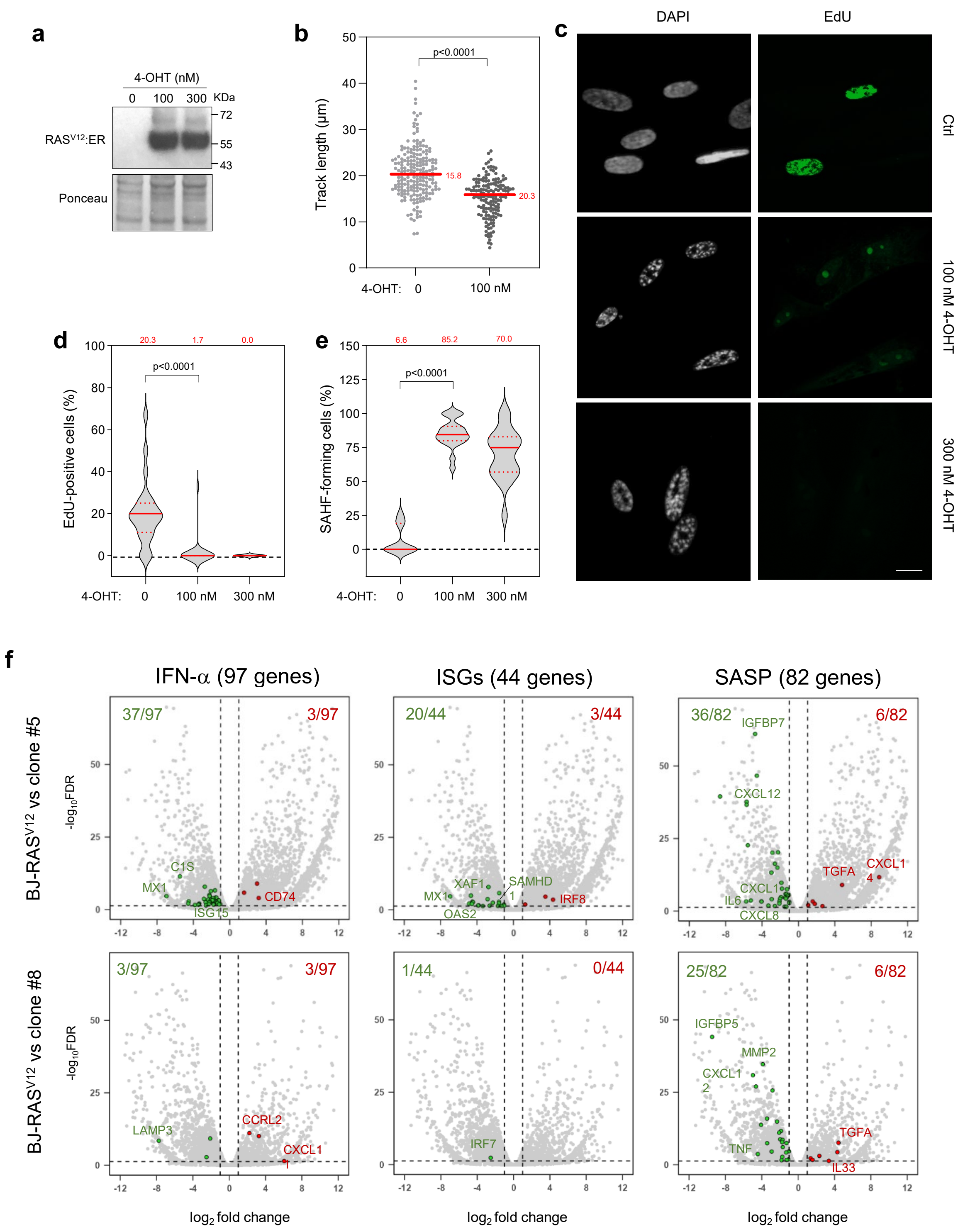

a
4-OHT (nM)
0 100 300 KDa
72
55
43
RASV12:ER
Ponceau
b
Track length (μm)
p<0.0001
15.8
20.3
4-OHT: 0 100 nM
c
DAPI
EdU
Ctrl
100 nM 4-OHT
300 nM 4-OHT
d
EdU-positive cells (%)
20.3 1.7 0.0
p<0.0001
4-OHT: 0 100 nM 300 nM
e
SAHF-forming cells (%)
6.6 85.2 70.0
p<0.0001
4-OHT: 0 100 nM 300 nM
f
IFN-α (97 genes)
ISGs (44 genes)
SASP (82 genes)
BJ-RASV12 vs clone #5
BJ-RASV12 vs clone #8
-log10FDR
37/97 3/97
C1S
MX1
ISG15
CD74
20/44 3/44
XAF1
MX1
OAS2
SAMHD1
IRF8
36/82 6/82
IGFBP7
CXCL12
CXCL1
IL6
CXCL8
TGFA
CXCL14
3/97 3/97
LAMP3
CCRL2
CXCL11
1/44 0/44
IRF7
25/82 6/82
IGFBP5
MMP2
CXCL12
TNF
TGFA
IL33
log2 fold change

Sup. Figure 1

Supplementary Figure legends

Supplementary Fig. 1 H-RAS^{V12} induction triggers senescence and induces the expression of ISG and SASP genes. a Human telomerase-immortalized lung fibroblasts hTERT-IMR90 harboring H-RAS^{V12} fused to an estrogen receptor were treated with different concentrations of 4-hydroxytamoxifen (4-OHT) for 6 days. Induction of H-RAS^{V12}:ER was detected by Western blotting. **b** Replication fork progression was monitored by DNA fiber spreading on day 6 after 4-OHT treatment. The median IdU+CldU track length is indicated in red. A minimum of 150 fibers were measured. A representative experiment is shown (n=3). The p value was determined using two-tailed Mann-Whitney rank sum test. **c** Inhibition of DNA replication by H-RAS^{V12} was quantified by EdU click chemistry. Representative images of EdU incorporation are shown (n=3). Scale bar: 10 µm. **d** Percentage of EdU-positive cells was quantified. The p value was determined using two-tailed Mann-Whitney rank sum test. A minimum of 200 cells were measured. A representative experiment is shown (n=2). **e** SAHF formation were quantified by DAPI staining on day 6 after 4-OHT treatment. $p < 0.0001$, two-tailed Mann-Whitney rank sum test. A representative experiment is shown (n=3). **f** Volcano plots of differentially expressed IFN-α, ISG and SASP genes in BJ-RAS^{V12} fibroblasts. Differential gene expression in senescent Dox-induced BJ-RAS^{V12} fibroblasts and in BJ-RAS^{V12} clones #5 and #8 is shown. Clone #5 escaped senescence by overexpressing Claspin and Timeless[11]. Clone #8 escaped senescence independently of Claspin and Timeless overexpression[11]. RNA-seq data (biological duplicates) were processed as indicated in Fig.1c.

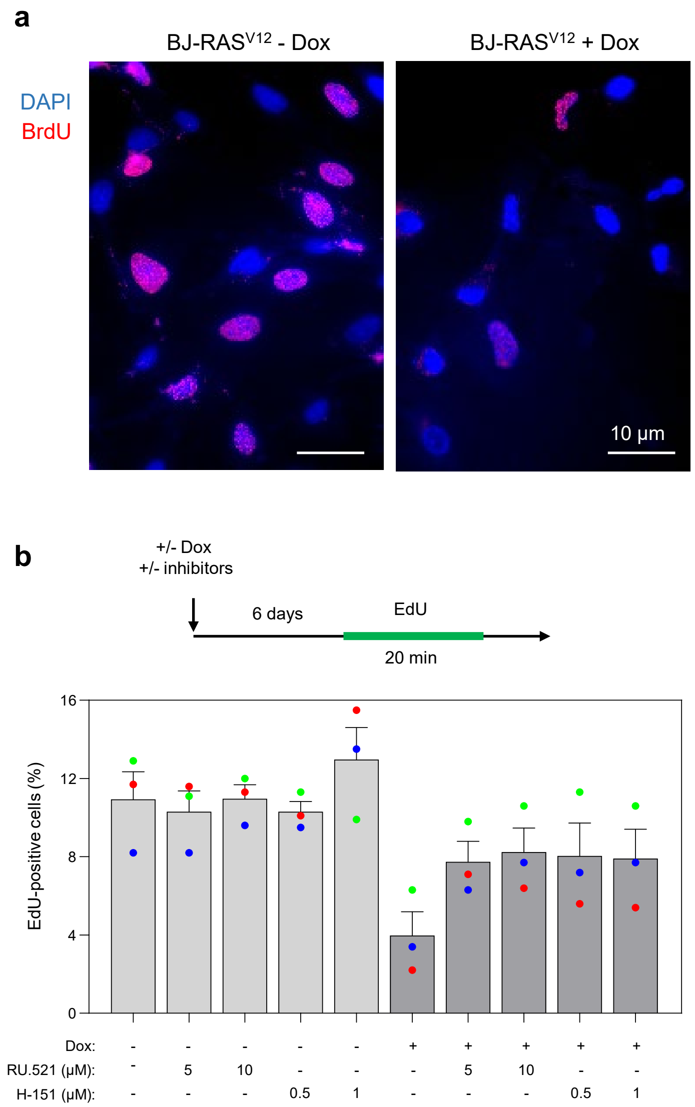

Supplementary Fig. 2 cGAS and STING inhibitors counteract RAS^{V12}-induced replication inhibition. a Representative images of BrdU incorporation upon RAS^{V12} induction in BJ fibroblasts. **b** BJ-RAS^{V12} fibroblasts were treated or not with 10 µg/ml doxycycline and with increasing doses of cGAS and STING inhibitors (RU.521 and H-151, respectively) for 6 days. The cells were then pulse labeled with 10 µM EdU for 20 minutes. The percentage of cells in S phase was analyzed using EdU click chemistry and flow cytometry. Mean and SEM are presented for three independent experiments.

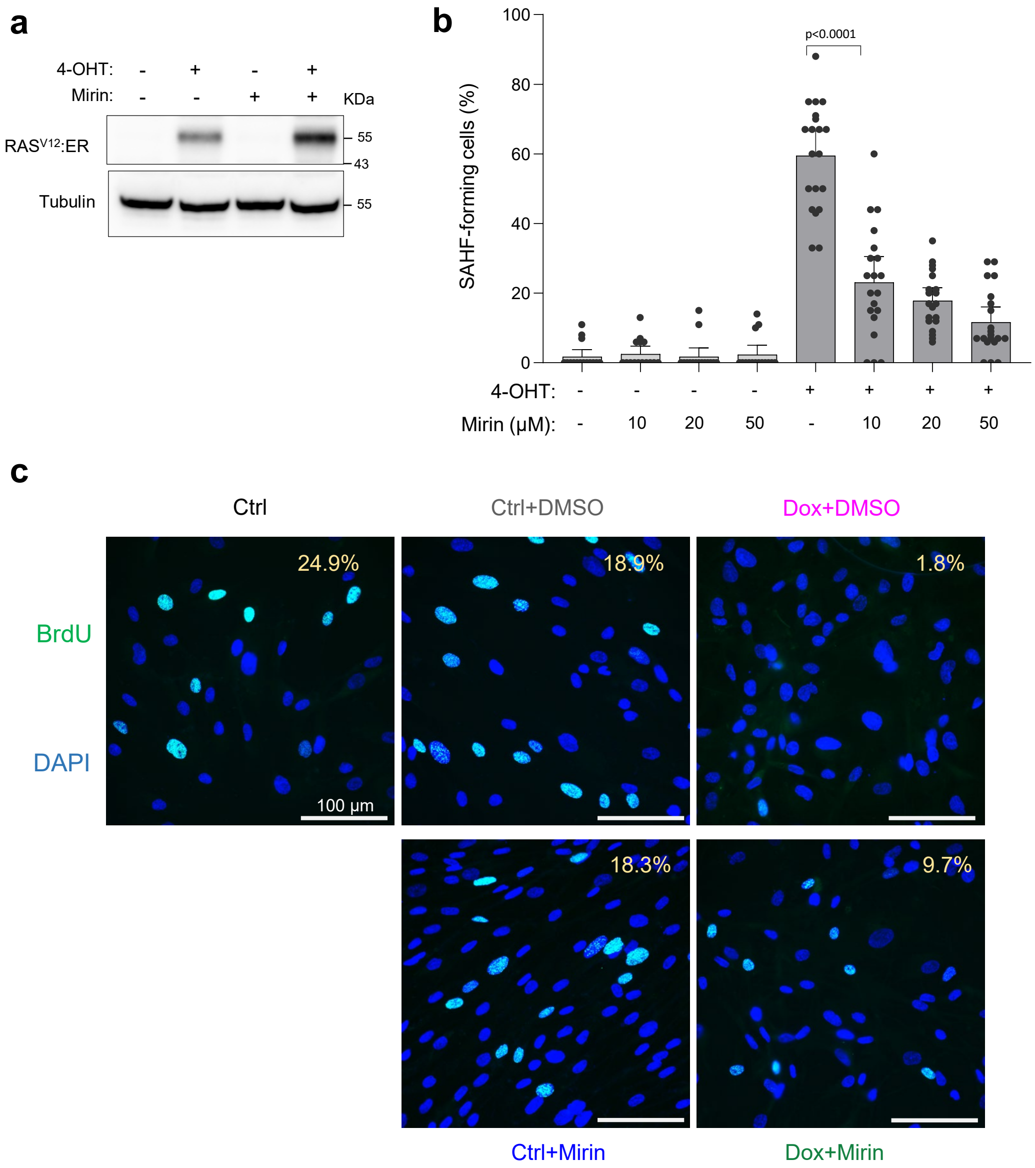

Supplementary Fig. 3 Mirin suppresses OIS in human fibroblasts. a IMR90-ER/RAS^{V12} fibroblasts were treated or not with 100 nM 4-hydroxytamoxifen (4-OHT) and 20 µM Mirin for 6 days. RAS^{V12}:ER expression was detected by Western blotting. **b** Mirin prevents the formation of RAS^{V12}-induced senescence-associated heterochromatin foci (SAHF). IMR90-ER/RAS^{V12} fibroblasts were treated or not with 100 nM 4-hydroxytamoxifen (4-OHT) and increasing doses of Mirin for 6 days. The percentage of SAHF was quantified by DAPI staining. $p < 0.0001$, two-tailed Mann-Whitney rank sum test. Ten images in each sample were collected. A minimum of 200 cells were monitored. A representative experiment is shown here (n=2). The p value was determined using two-tailed Mann-Whitney rank sum test. **c** Analysis of BrdU incorporation. Cells were treated with Mirin five days post-RAS^{V12} induction (+ Dox) for three days. DAPI is in blue, and BrdU is in green. Scale bar is 100 µm. The percentage of BrdU positive-cells is indicated.

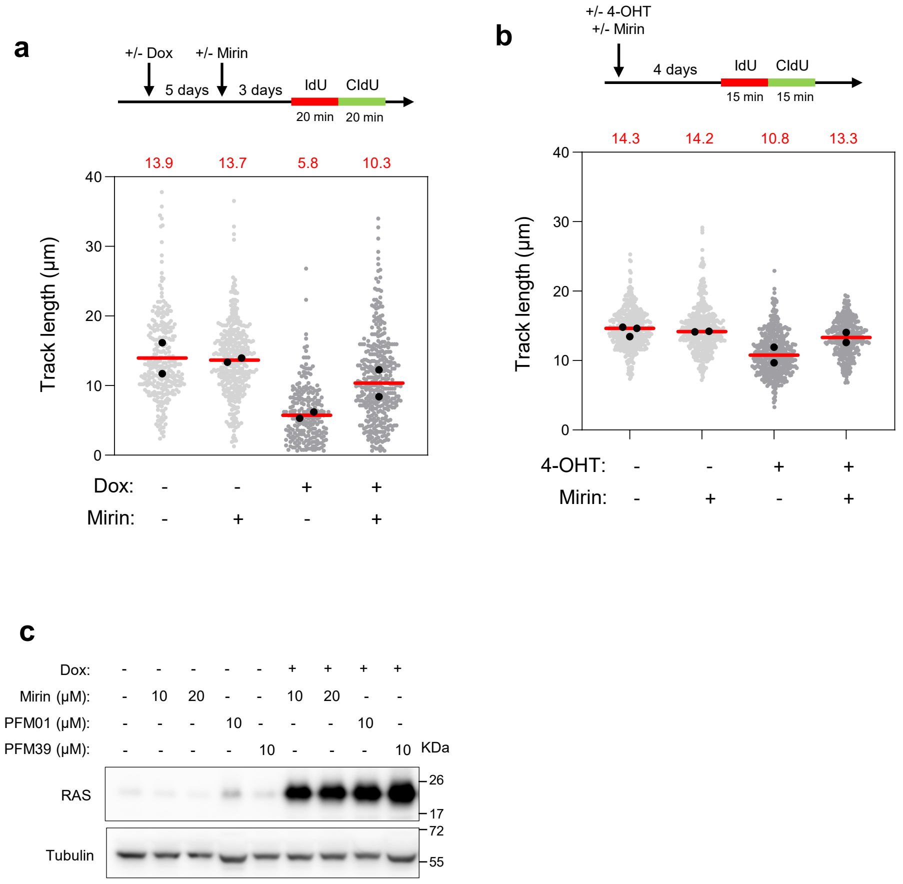

Supplementary Fig. 4 Mirin suppresses replication stress in BJ-RAS^{V12} fibroblasts. a DNA fiber analysis of fork progression in BJ-RAS^{V12} cells treated or not with Dox for 8 days and treated with or without 10 µM Mirin for the last 3 days of Dox induction. The median of IdU+CldU track length of two independent experiments is shown in red. A minimum of 150 fibers were measured in each sample and each biological replicates (n=2). **b** Mirin restores RAS^{V12}-induced fork slowing in IMR90-ER/RAS^{V12} fibroblasts. Cells were treated with or without 100 nM 4-hydroxytamoxifen and 20 µM Mirin for 4 days. Replication fork progression was measured by DNA fiber spreading as described. The median of IdU+CldU track length of two independent experiments is indicated in red. A minimum of 150 fibers were measured in each sample and each biological replicates (n=2). **c** BJ-RAS^{V12} were treated with or without 10 µg/ml doxycycline in the presence or absence of MRE11 inhibitors, Mirin, PFM01 or PFM39. The expression of RAS^{V12} was detected by Western blotting. Alpha-tubulin was included as a loading control.

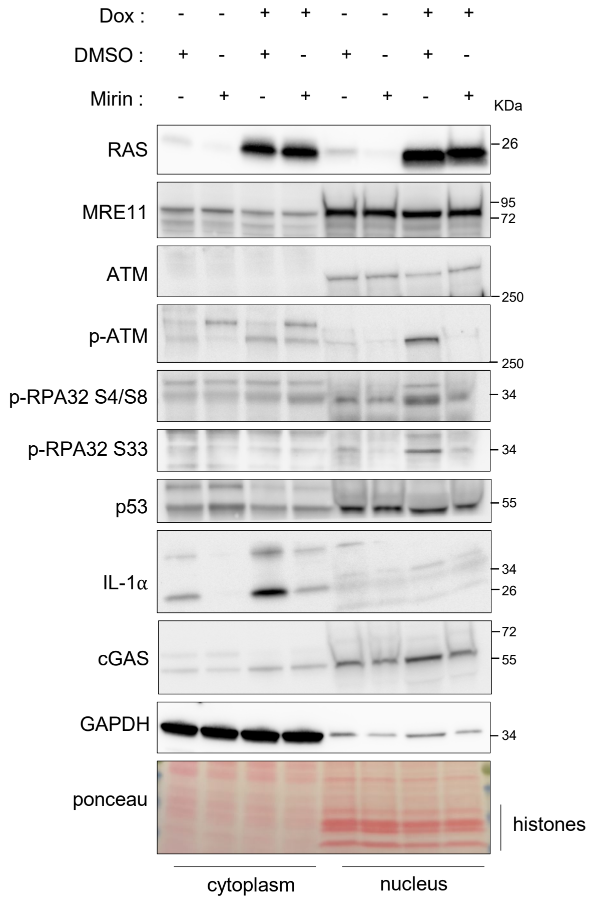

Supplementary Fig. 5 Effect of Mirin on IL1-α expression in BJ-RAS^{V12} fibroblasts. Protein levels of the SASP factor IL1-α and of other indicated factors were analyzed by Western blot in BJ fibroblasts overexpressing (+Dox) or not (-Dox) RAS^{V12} and treated or not with 10 µM Mirin for 8 days. Cytosolic and nuclear fractions were collected and processed for immunoblotting assay. GAPDH and Ponceau staining are shown as fractionation and loading controls.

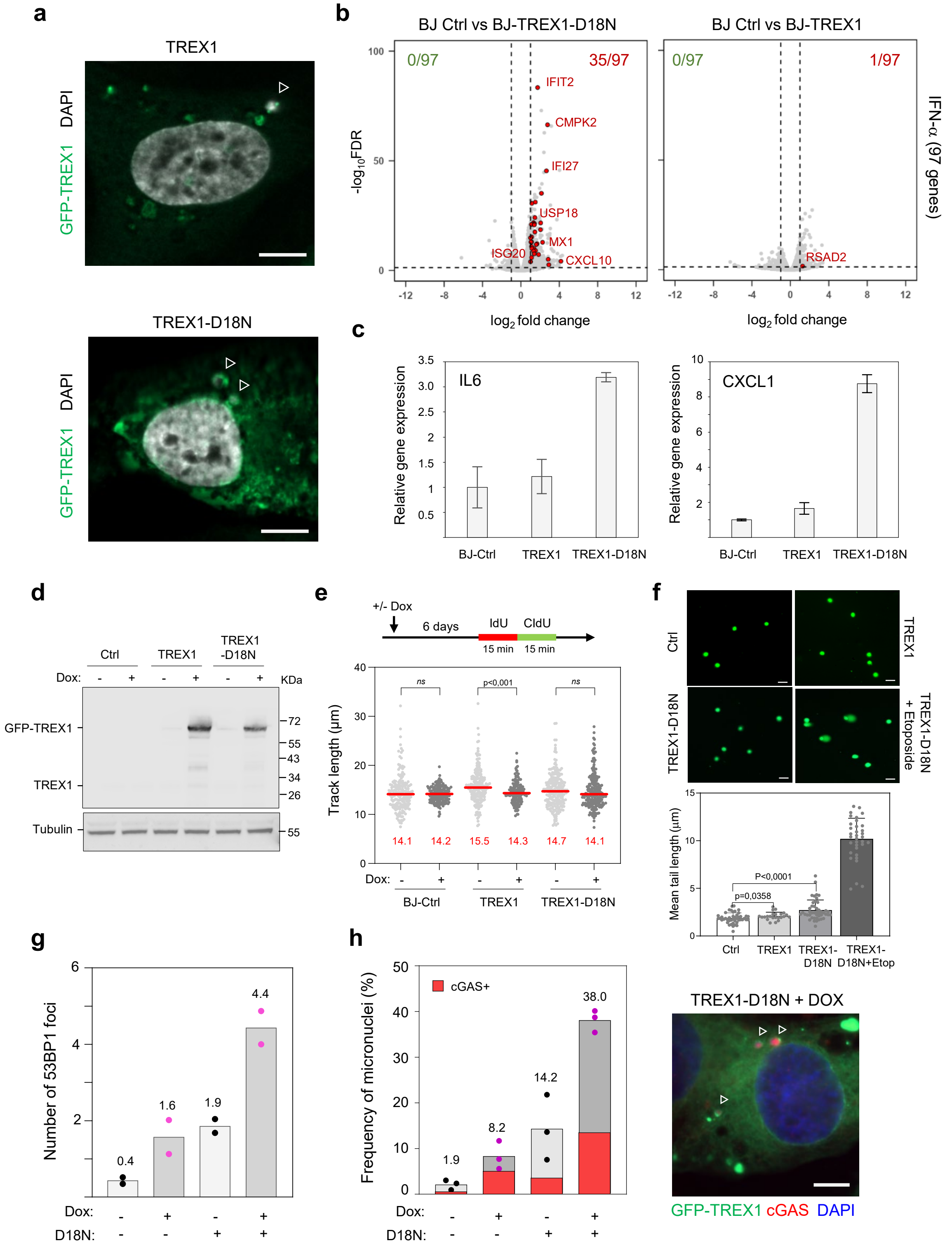

a
TREX1
GFP-TREX1 DAPI
TREX1-D18N
b
BJ Ctrl vs BJ-TREX1-D18N
BJ Ctrl vs BJ-TREX1
0/97
35/97
1/97
IFIT2
CMPK2
IFI27
USP18
MX1
ISG20
CXCL10
RSAD2
-log10FDR
log2 fold change
IFN-α (97 genes)
c
IL6
CXCL1
Relative gene expression
BJ-Ctrl
TREX1
TREX1-D18N
d
Ctrl
TREX1
TREX1 -D18N
Dox:
KDa
GFP-TREX1
TREX1
Tubulin
e
+/- Dox
6 days
IdU
CldU
15 min
15 min
Track length (µm)
ns
p<0,001
14.1
14.2
15.5
14.3
14.7
BJ-Ctrl
TREX1
TREX1-D18N
f
Ctrl
TREX1
TREX1-D18N
TREX1-D18N + Etoposide
Mean tail length (µm)
P<0,0001
p=0,0358
TREX1-D18N
TREX1-D18N+Etop
g
Number of 53BP1 foci
0.4
1.6
1.9
4.4
D18N:
h
cGAS+
Frequency of micronuclei (%)
1.9
8.2
14.2
38.0
TREX1-D18N + DOX
GFP-TREX1 cGAS DAPI

Sup. Figure 6

Supplementary Fig. 6 TREX1 regulates the IFN response in BJ-RAS ^{V12} fibroblasts. **a** GFP-TREX1 colocalizes with micronuclei. Representative images from two independent experiments are shown. Scale bar: 5 μm. **b** Volcano plots of differentially expressed IFN-α response genes in non-induced BJ-RAS cells (BJ-Ctrl) compared to cells overexpressing either TREX1 or TREX1-D18N. RNA-seq samples were processed and analyzed as indicated in Fig. 1c. **c** RT-qPCR analysis of *IL6* and *CXCL1* gene expression in indicated cell lines. Mean $\pm$ SD of one representative experiment is shown (n=3). **d** Parental BJ-hTERT fibroblasts or BJ-hTERT harboring tetracycline-inducible TREX1 or TREX1-D18N were treated or not with 4 μg/ml doxycycline. The expression of EGFP-TREX1 was detected by Western blotting. Alpha-tubulin was included as a loading control. **e** Overexpression of TREX1 or TREX1-D18N does not affect replication fork progression. BJ fibroblasts expressing tetracycline-inducible TREX1 or the dominant negative mutant D18N were treated or not with 4 μg/ml doxycycline for 6 days. Replication fork progression was measured by DNA fiber spreading. Median IdU+CldU track length is shown in red. The p value was determined using two-tailed Mann-Whitney rank sum test. *ns*: not significant. A representative experiment is shown (n=3). **f** Overexpression of TREX1 or TREX1-D18N induces detectable levels of chromosome breaks. BJ fibroblasts expressing tetracycline-inducible TREX1 or the dominant negative mutant D18N were treated or not with 4 μg/ml doxycycline for 8 days followed by alkaline comet assay. Treatment with 20 μM etoposide for 5 hours was included as a positive control. Representative images from two independent experiments are shown. Scale bar: 20 μm. At least 30-50 nuclei were measured for each sample. Mean tail length and SD are shown (n=2). The p values were determined using two-tailed Mann-Whitney rank sum test. **g** Number of 53BP1 foci in cells overexpressing RAS^{V12} and the dominant negative mutant of TREX1 (TREX1-D18N; n=2). **h** Frequency of micronuclei in cells overexpressing RAS^{V12} and/or TREX1-D18N (n=3). The localization of cGAS was determined by immunofluorescence. The frequency of cGAS+ micronuclei in BJ cells overexpressing RAS^{V12} and/or the TREX1-D18N mutant is shown in red (n=2). A representative image showing cGAS-positive micronuclei (arrowheads) is shown. Scale bar is 5 μm.

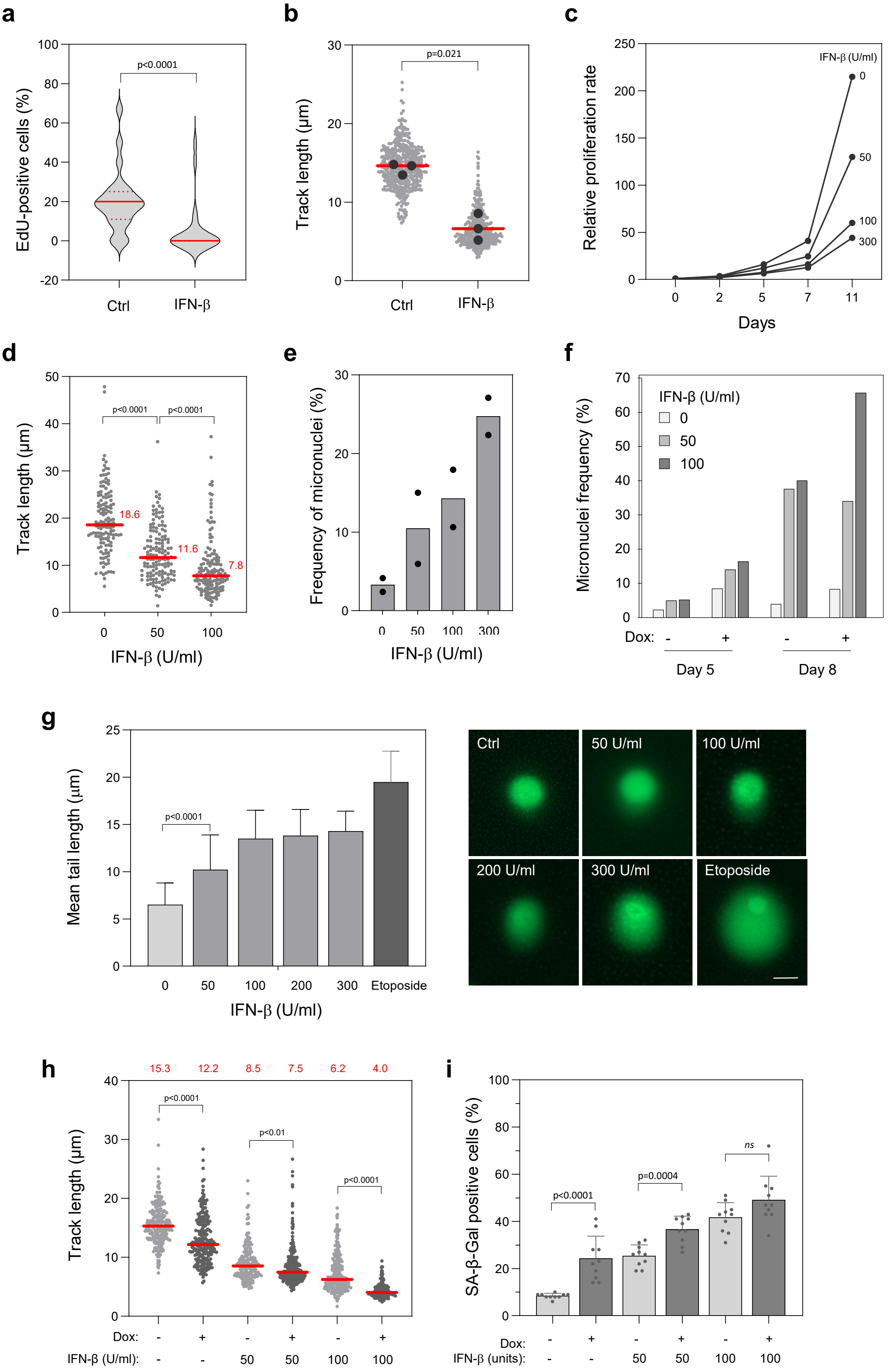
a
EdU-positive cells (%)
p<0.0001
Ctrl
IFN-β
b
Track length (µm)
p=0.021
Ctrl
IFN-β
c
Relative proliferation rate
IFN-β (U/ml)
0
50
100
300
Days
d
Track length (µm)
p<0.0001
p<0.0001
18.6
11.6
7.8
IFN-β (U/ml)
e
Frequency of micronuclei (%)
IFN-β (U/ml)
f
Micronuclei frequency (%)
IFN-β (U/ml)
0
50
100
Dox:
Day 5
Day 8
g
Mean tail length (µm)
p<0.0001
0
50
100
200
300
Etoposide
IFN-β (U/ml)
Ctrl
50 U/ml
100 U/ml
200 U/ml
300 U/ml
Etoposide
h
15.3
12.2
8.5
7.5
6.2
4.0
Track length (µm)
p<0.0001
p<0.01
p<0.0001
Dox:
IFN-β (U/ml):
i
SA-β-Gal positive cells (%)
p<0.0001
p=0.0004
ns
Dox:
IFN-β (units):

Sup. Figure 7

Supplementary Fig. 7 IFN-β induces growth inhibition, DNA breaks and micronuclei. Interferon-β treatment inhibits cell proliferation and induces senescence, fork slowdown and micronuclei formation in hTERT-immortalized IMR90 fibroblasts and RPE-1 cells. **a** IMR90 fibroblasts were treated with 50 U/ml of IFN-β for 6 days. Inhibition of DNA replication by IFN-β was monitored by EdU click chemistry. Percentage of EdU-positive cells was quantified. A representative experiment is shown (n=2). $p < 0.0001$, two-tailed Mann-Whitney rank sum test. **b** Replication fork progression was monitored by DNA fiber spreading on day 6 in IMR90 cells treated or not with 50 U/ml IFN-β. The median of 3 independent experiments is indicated in red, two-tailed paired t-test. **c** Human telomerase-immortalized retinal pigment epithelial hTERT-RPE-1 cells were treated or not with increasing doses of IFN-β. Cell number was counted at the indicated time points. A summary of three independent experiments is shown. **d** Replication fork progression was monitored by DNA fiber spreading 8 days after IFN-β treatment. Median IdU+CldU track length is indicated in red (n=1). $p < 0.0001$, two-tailed Mann-Whitney rank sum test. **e** Frequency of micronuclei in cells supplemented with either 50 or 100 U/ml IFN-β for 8 days. The mean of two independent experiments is shown. **f** Frequency of micronuclei in BJ-RAS^{V12} cells treated or not with 10 μg/ml Doxycycline and IFN-β for 5 to 8 days. A representative experiment is shown. **g** IFN-β treatment induces chromosome breaks in hTERT-immortalized RPE-1 cells. Human hTERT-RPE-1 cells were treated for 6 days with increasing doses of IFN-β. Comet assay was performed under alkaline conditions and tail length was measured using the MetaMorph software. At least 30-50 nuclei were measured for each sample. Mean tail length and SD are shown (n=1). $p < 0.0001$, two-tailed Mann-Whitney rank sum test. **h** IFN-β significantly enhances RAS^{V12}-induced fork slowdown. BJ-RAS^{V12} fibroblasts were treated or not with 10 μg/ml doxycycline and IFN-β for 6 days. Replication fork progression was measured by DNA fiber spreading. Median IdU+CldU track length is indicated in red. The p values were determined using two-tailed Mann-Whitney rank sum test. A representative experiment from two biological replicates is presented. **i** IFN-β enhances RAS^{V12}-induced senescence. BJ-RAS^{V12} fibroblasts were treated or not with 10 μg/ml doxycycline and IFN-β for 6 days. SA-b-gal activity was stained. Percentage of SA-b-gal-positive cells was scored. A representative experiment is shown (n=2). The p values were determined using two-tailed Mann-Whitney rank sum test. *ns*: non-significant.

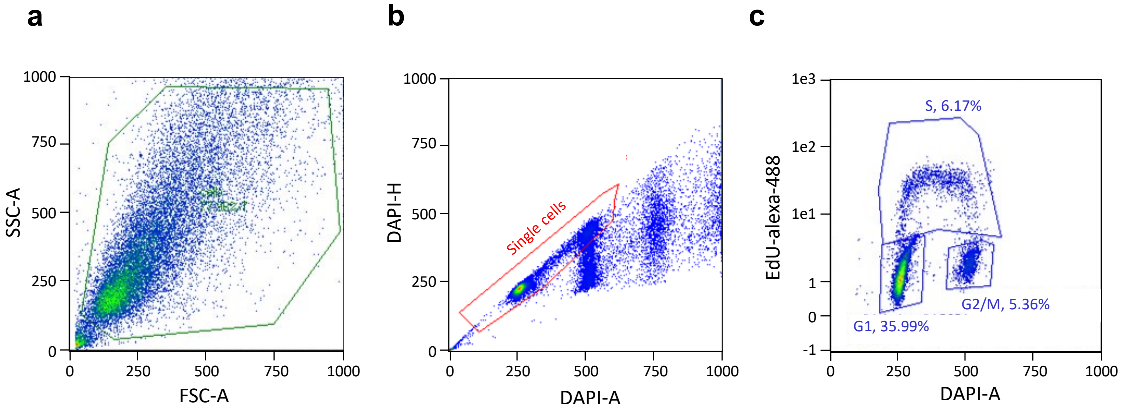

Supplementary Fig. 8 Flow cytometry gating strategy. a Selection of viable cells according to FSC and SSC. **b** Single cell gating based on the DNA content. **c** Percentage of cells in S phase was quantified according to the EdU-Alexa-488 fluorescence.
