# Supplementary material for: MRE11 and TREX1 control senescence by coordinating replication stress and interferon signaling
Source: Nat Commun. 2024 Jun 26;15:5423. doi: 10.1038/s41467-024-49740-w (PMC11208572; doi:10.1038/s41467-024-49740-w)
Supplement: Supplementary file 3 — Reporting Summary [file 41467_2024_49740_MOESM3_ESM.pdf]

nature portfolio

Corresponding author(s): Philippe Pasero and Yea-Lih Lin
Last updated by author(s): Apr. 21, 2024

Reporting Summary

Nature Portfolio wishes to improve the reproducibility of the work that we publish. This form provides structure for consistency and transparency in reporting. For further information on Nature Portfolio policies, see our Editorial Policies and the Editorial Policy Checklist.

Statistics

For all statistical analyses, confirm that the following items are present in the figure legend, table legend, main text, or Methods section.

n/a	Confirmed	
☐	☒	The exact sample size (n) for each experimental group/condition, given as a discrete number and unit of measurement
☒	☐	A statement on whether measurements were taken from distinct samples or whether the same sample was measured repeatedly
☐	☒	The statistical test(s) used AND whether they are one- or two-sided<br>*Only common tests should be described solely by name; describe more complex techniques in the Methods section.*
☒	☐	A description of all covariates tested
☒	☐	A description of any assumptions or corrections, such as tests of normality and adjustment for multiple comparisons
☐	☒	A full description of the statistical parameters including central tendency (e.g. means) or other basic estimates (e.g. regression coefficient) AND variation (e.g. standard deviation) or associated estimates of uncertainty (e.g. confidence intervals)
☐	☒	For null hypothesis testing, the test statistic (e.g. F, t, r) with confidence intervals, effect sizes, degrees of freedom and P value noted<br>*Give P values as exact values whenever suitable.*
☒	☐	For Bayesian analysis, information on the choice of priors and Markov chain Monte Carlo settings
☒	☐	For hierarchical and complex designs, identification of the appropriate level for tests and full reporting of outcomes
☒	☐	Estimates of effect sizes (e.g. Cohen's d, Pearson's r), indicating how they were calculated

Our web collection on statistics for biologists contains articles on many of the points above.

Software and code

Policy information about availability of computer code

Data collection	RNA-seq used Spliced Transcripts Alignment to a Reference (STAR).
Data analysis	The DNA fibers were measured by MetaMorph Microscopy Automation and Image Analysis Software (Molecular Devices) and statistical analysis was performed with GraphPad Prism 8 (GraphPad Software). The quality of sequencing data was assessed with FastQC (http://www.bioinformatics.babraham.ac.uk/projects/fastqc). Flow cytometry data were analyzed using FlowJo 10 (LLC).

For manuscripts utilizing custom algorithms or software that are central to the research but not yet described in published literature, software must be made available to editors and reviewers. We strongly encourage code deposition in a community repository (e.g. GitHub). See the Nature Portfolio guidelines for submitting code & software for further information.

Data

Policy information about availability of data

All manuscripts must include a data availability statement. This statement should provide the following information, where applicable:

- Accession codes, unique identifiers, or web links for publicly available datasets
- A description of any restrictions on data availability
- For clinical datasets or third party data, please ensure that the statement adheres to our policy

Source data are provided with this paper. The datasets generated during and/or analyzed during the current study are available from the corresponding author on reasonable request. The NGS datasets generated and analyzed during the current study are available in the BioSample database , Temporary SubmissionID: SUB13958437

Research involving human participants, their data, or biological material

Policy information about studies with human participants or human data. See also policy information about sex, gender (identity/presentation), and sexual orientation and race, ethnicity and racism.

Reporting on sex and gender	Non applicable
Reporting on race, ethnicity, or other socially relevant groupings	Non applicable
Population characteristics	Non applicable
Recruitment	Non applicable
Ethics oversight	Non applicable

Note that full information on the approval of the study protocol must also be provided in the manuscript.

Field-specific reporting

Please select the one below that is the best fit for your research. If you are not sure, read the appropriate sections before making your selection.

☒ Life sciences ☐ Behavioural & social sciences ☐ Ecological, evolutionary & environmental sciences

For a reference copy of the document with all sections, see nature.com/documents/nr-reporting-summary-flat.pdf

Life sciences study design

All studies must disclose on these points even when the disclosure is negative.

Sample size	Sample size was determined according to field standards. For DNA fiber spreading, at least 150 fibers were measured. For comet assay, tail length was measured in 30-50 tailing cells. Mean fluorescence intensity (MFI) quantification was performed on more than 400 cells.
Data exclusions	No data were excluded.
Replication	RAN-Seq datasets were reproduced at least twice with the same method. Most of the experiments were reproduced at least twice. All attempts at replication were successful.
Randomization	Randomization is not relevant to our study. Each sample has a specific treatment. It is not possible to randomize samples between different treatments.
Blinding	For DNA fiber assays, sample treatments were given a number instead of naming an exact treatment.

Reporting for specific materials, systems and methods

We require information from authors about some types of materials, experimental systems and methods used in many studies. Here, indicate whether each material, system or method listed is relevant to your study. If you are not sure if a list item applies to your research, read the appropriate section before selecting a response.

Materials & experimental systems

n/a	Involved in the study	
☐	☒	Antibodies
☐	☒	Eukaryotic cell lines
☒	☐	Palaeontology and archaeology
☒	☐	Animals and other organisms
☒	☐	Clinical data
☒	☐	Dual use research of concern
☒	☐	Plants

Methods

n/a	Involved in the study	
☒	☐	ChIP-seq
☐	☒	Flow cytometry
☒	☐	MRI-based neuroimaging

Antibodies

Antibodies used	anti-RAS (BD, ref 610002); anti-MRE11 (Novus, NBS100-142); anti-cGAS (Cell Signaling, #15102); anti-p53 (Santa Cruz, clone DO7 sc-47698); Following antibodies were purchased from Abcam: anti-ATM (ab183324); anti-phospho S1981 ATM (ab81292); anti-IL1-a (ab9614); anti-ISG15 (ab13346); anti-TREX1 (ab185228); Rabbit anti-Phospho RPA32 (S33) and anti-Phospho RPA32 (S4/S8) were

	from Bethyl (A300-246A and A300-245A, respectively). Mouse anti-BrdU (BD 347580); Rat anti-BrdU (Eurobio clone BU1/75); Goat anti-rat Alexa 488 (Molecular Probes, A11006); Goat anti-mouse IgG1Alexa 546 (Molecular Probes, A21123); Mouse anti-ssDNA (auto anti-ssDNA, DSHB); Goat anti-mouse IgG2a Alexa 647 (Molecular Probes, A21241).
Validation	anti-RAS (1/500), anti-MRE11 (1/500), anti-cGAS (1/500), anti-p53 (1/500). anti-ATM (1:500, ab183324), anti-phospho S1981 ATM (1/500, ab81292), anti-IL1-a (1/500, ab9614), anti-ISG15 (1/1000, ab13346), anti-TREX1 (1:1000, ab185228). Rabbit anti-Phospho RPA32 (S33) and anti-Phospho RPA32 (S4/S8) were from Bethyl (used at 1/500, A300-246A and A300-245A, respectively). Mouse anti-BrdU to detect IdU (1/200, BD 347580), Rat anti-BrdU to detect CldU (1/100, Eurobio clone BU1/75). Secondary antibody mix: Goat anti-rat Alexa 488 (1/200 PBS/T, Molecular Probes, A11006); Goat anti-mouse IgG1Alexa 546 (1/200 PBS/T, Molecular Probes, A21123). The ssDNA is detected with Anti-ssDNA (1/200) and then the goat anti-Mouse IgG2a Alexa 647 (1/200).

Eukaryotic cell lines

Policy information about cell lines and Sex and Gender in Research

Cell line source(s)	Normal human fibroblasts BJ-hTERT were a gift of Dr. D. Peeper (The Netherlands Cancer Institute, Amsterdam). IMR90-ER/RASV12 cells were a gift of Masashi Narita (Young et al. 2010). RPE1-hTERT cells were from ATCC (CRL-4000).
Authentication	None of the cell lines have been authenticated by Standards for Cell Line Authentification (Almeida et al. 2016). However, RPE1-hTERT cells have been purchased from ATCC and authenticated by this organization with certificates.
Mycoplasma contamination	Mycoplasma testing by qPCR was performed periodically to ensure that the cell lines are mycoplasma free.
Commonly misidentified lines (See ICLAC register)	No commonly misdefined cell lines were used in the study.

Plants

Seed stocks	non applicable
Novel plant genotypes	non applicable
Authentication	non applicable

Flow Cytometry

Plots

Confirm that:

☒ The axis labels state the marker and fluorochrome used (e.g. CD4-FITC).

☒ The axis scales are clearly visible. Include numbers along axes only for bottom left plot of group (a 'group' is an analysis of identical markers).

☒ All plots are contour plots with outliers or pseudocolor plots.

☒ A numerical value for number of cells or percentage (with statistics) is provided.

Methodology

Sample preparation	Cells were pulse-labeled with EdU and fixed in 1% PFA. EdU click chemistry was performed prior to flow cytometry analyses.
Instrument	MACSQuant analyser
Software	FlowJo (LLC)
Cell population abundance	10.000 cells were collected for the analyses.
Gating strategy	Cellular integrity was first selected according to the SSC and FSC. Single cell population was then gated according to the DNA content. The fluorescence intensity increase was further gated as cells in S phase, using non-EdU-labeled cells as a negative control.

☒ Tick this box to confirm that a figure exemplifying the gating strategy is provided in the Supplementary Information.
